# Delayed Autoimmune Hemolytic Anemia After Remission in Non-Hodgkin Lymphoma

**DOI:** 10.7759/cureus.106120

**Published:** 2026-03-30

**Authors:** Nicholas Azinge, Tigabu Bishaw, Raghav Veluri, Mekdem Bisrat, Anand Deonarine, Ahmed Ali

**Affiliations:** 1 Internal Medicine, Howard University Hospital, Washington, DC, USA

**Keywords:** autoimmune hemolytic anemia, autoimmunity, hemolysis, long-term surveillance, non-hodgkin lymphoma, residual disease

## Abstract

A 46-year-old man with non-Hodgkin lymphoma (NHL) in remission since 2014 presented with progressive dizziness, fatigue, and dark-colored urine. Laboratory workup revealed hemoglobin levels of 5.9 g/dL, elevated lactate dehydrogenase (LDH), hyperbilirubinemia, low haptoglobin, and a positive direct Coombs test for IgG, C3, and poly-specific antibodies, confirming warm autoimmune hemolytic anemia (wAIHA). Immunoglobulin profiling showed low IgM and IgA with high-normal IgG, suggesting chronic immune dysregulation, whereas abdominal imaging revealed mesenteric calcifications, raising concern for residual lymphomatous tissue potentially driving ongoing autoimmune activation. Despite corticosteroids and intravenous immunoglobulin (IVIG), persistent hemolysis necessitated four units of packed red blood cell (PRBC) transfusions and escalation to rituximab, after which gradual clinical stabilization was achieved, highlighting the refractory nature of wAIHA in the setting of previous lymphoproliferative malignancy. Serial monitoring of hemoglobin, reticulocyte count, and LDH throughout the clinical course demonstrated dynamic trends consistent with partial but meaningful immunosuppression, underscoring the value of longitudinal laboratory surveillance in guiding therapeutic escalation decisions. The presence of mesenteric calcifications, combined with a skewed immunoglobulin profile, provided important structural and immunologic clues to the underlying pathophysiology, illustrating how multimodal diagnostic evaluation can reveal occult contributors to delayed autoimmune phenomena even in the absence of overt malignancy recurrence. This case underscores the importance of recognizing delayed AHA in NHL survivors and the necessity of long-term hematologic surveillance, periodic cross-sectional imaging, and a low threshold for multidisciplinary consultation even after prolonged remission.

## Introduction

Autoimmune hemolytic anemia (AIHA) is a disorder characterized by immune-mediated destruction of red blood cells (RBCs) through autoantibodies directed against erythrocyte surface antigens. It is broadly classified into warm, cold, and mixed subtypes based on the thermal reactivity of the offending antibodies [1,2]. It may arise idiopathically or secondary to underlying conditions, most notably lymphoproliferative disorders such as non-Hodgkin lymphoma (NHL), chronic lymphocytic leukemia (CLL), autoimmune diseases, viral infections, and certain medications [2,3]. The association between NHL and AIHA is well established, reflecting immune dysregulation driven by malignant B-cell clones capable of producing pathologic autoantibodies targeting host erythrocytes [2,4,5]. Although AIHA most frequently presents concurrently with active or relapsed lymphoma, delayed onset occurring years after achieving complete remission represents a rare and diagnostically challenging clinical entity that is increasingly recognized in the literature [1,2]. Clinicians must therefore maintain a high index of suspicion for AIHA in any patient with a previous lymphoproliferative malignancy who presents with unexplained anemia, regardless of the duration of remission [3,4].

The pathogenesis of late-onset AIHA in the setting of previous lymphoma remains incompletely understood but is believed to involve a spectrum of overlapping mechanisms [2]. Persistent dysregulation of B-cell immunoregulatory networks, failure of central and peripheral immune tolerance, and treatment-induced perturbations of the immune microenvironment may collectively predispose susceptible individuals to autoantibody production long after therapy completion [3,6]. Additionally, residual subclinical lymphomatous tissue evading standard surveillance may serve as an ongoing source of immune stimulation, driving chronic autoimmune activation even in the absence of overt disease recurrence [7]. Recognizing the temporal trends of key hematologic and immunologic parameters - including hemoglobin trajectory, reticulocyte count, lactate dehydrogenase (LDH), indirect bilirubin, haptoglobin, and direct antiglobulin testing - is essential not only for establishing an early and accurate diagnosis but also for monitoring treatment response and guiding escalation of therapy [1,4,6]. A structured and multidisciplinary approach integrating hematology, oncology, and immunology expertise is therefore critical to optimizing outcomes in this complex and underrecognized patient population [4,6,8].

## Case presentation

The patient, a 46-year-old man, arrived at the emergency department reporting a four-day history of progressively worsening dizziness, palpitations, and orthostatic fatigue that significantly impaired his ability to perform daily activities. He additionally described generalized pallor and a two-week history of dark-colored urine, raising concern for hemoglobinuria and prompting urgent hematologic evaluation. He denied any accompanying fever, night sweats, chest pain, unintentional weight loss, or urinary symptoms that might suggest an alternative or concurrent diagnosis. His past medical history was significant for NHL diagnosed and treated with systemic chemotherapy and RBC transfusions in 2014, after which he achieved and maintained complete clinical remission. Notably, he had no documented history of smoking, illicit drug use, or previous autoimmune disease and was not on any medications known to cause drug-induced hemolytic anemia at the time of presentation.

**Figure 1:**
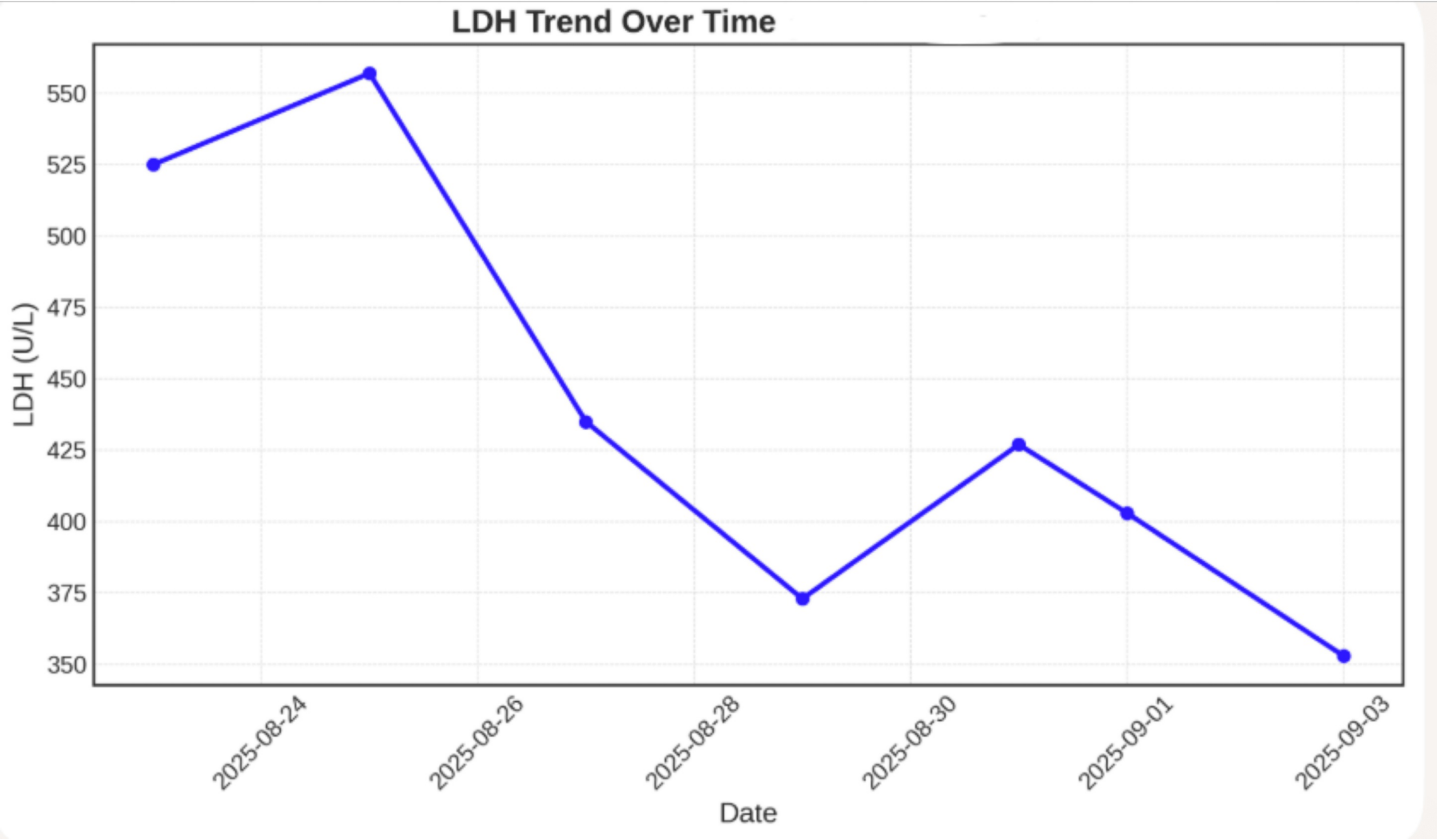
LDH activity over the course of treatment LDH: lactate dehydrogenase

**Figure 2:**
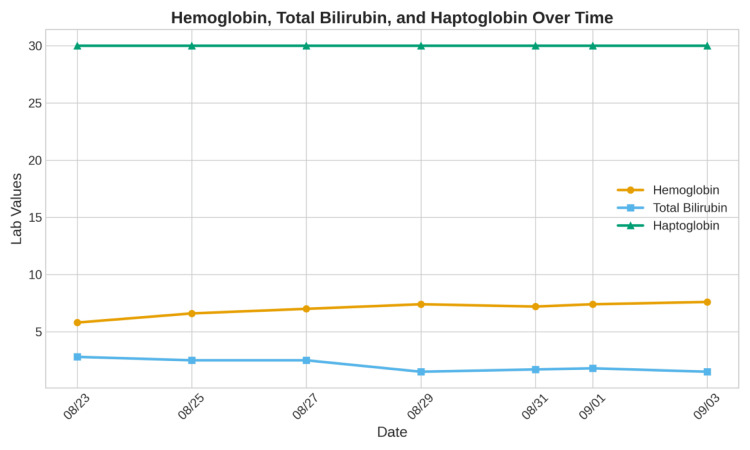
Blood workup results over the course of treatment

On physical examination, the patient appeared fatigued and mildly distressed. The most clinically significant positive findings were notable conjunctival pallor, consistent with severe underlying anemia, and resting tachycardia with a heart rate of 115 beats per minute, likely representing a compensatory physiologic response to critically reduced oxygen-carrying capacity. The remainder of the physical examination was unremarkable, with no palpable lymphadenopathy in the cervical, axillary, or inguinal regions and no hepatosplenomegaly appreciated on abdominal examination. Laboratory evaluation revealed a critically low hemoglobin of 5.9 g/dL, with supporting evidence of active hemolysis, including elevated LDH at 515 U/L, increased total bilirubin at 2.8 mg/dL, and markedly low haptoglobin below 30 mg/dL. The LDH trend over time is shown in Figure 1, and the patient's hemoglobin, total bilirubin, and haptoglobin over time are shown in Figure 2.

The direct Coombs test was positive for IgG, C3, and poly-specific antibodies, firmly confirming immune-mediated hemolysis, whereas immunoglobulin profiling demonstrated low IgM and IgA with high-normal IgG, a pattern suggestive of chronic immune dysregulation or ongoing immune activation. Markedly elevated ferritin at 1246.5 ng/mL and serum iron at 237 μg/dL further indicated iron overload, most likely attributable to chronic hemolysis rather than transfused iron accumulation, given the absence of a chronic transfusion history in this patient. The patient's investigations conducted during the course of his treatment are listed in Table 1.

**Table 1:** Initial blood work results The laboratory findings present a clear picture of severe anemia, with critically low hemoglobin levels of 5.9 g/dL secondary to active hemolysis. Supporting evidence of hemolysis includes elevated lactate dehydrogenase (LDH) at 515 U/L, increased total bilirubin at 2.8 mg/dL, and a markedly low haptoglobin level.

Test	Result	Reference range/interpretation
Hemoglobin	5.9 g/dL	Low
Lactate dehydrogenase (LDH)	515 U/L	Elevated
Total bilirubin	2.8 mg/dL	Elevated
Reticulocyte count	0.51%	Low
Haptoglobin	<30 mg/dL	Low
Direct Coombs test	Positive for IgG, C3, and poly-specific antibodies	Suggestive of autoimmune hemolysis
Immunofixation-IgM	31 mg/dL	Low
Immunofixation-IgG	1565 mg/dL	High-normal
Immunofixation-IgA	31 mg/dL	Low-normal
Ferritin	1246.5 ng/mL	Elevated
Serum iron	237 μg/dL	Elevated
Vitamin B12	307 pg/mL	Normal

**Figure 3:**
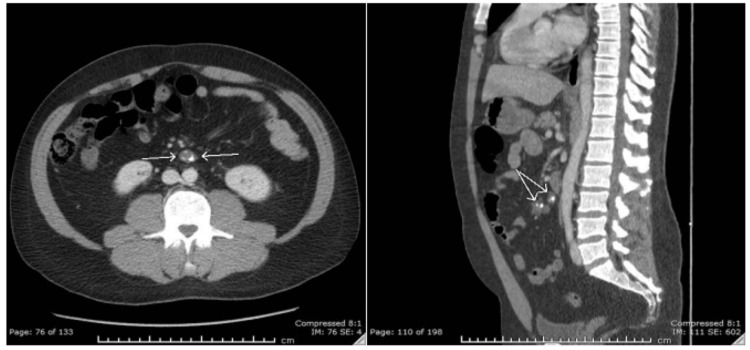
CT abdomen/pelvis Two mesenteric soft tissue foci with dystrophic calcifications (2.3 cm and 1.6 cm), possibly residual lymphoma or fat necrosis, are observed. White arrows on axial (left) and sagittal (right) views indicate calcified mesenteric foci. CT: computed tomography

An incidental discovery of mesenteric calcifications on abdominal imaging in this patient raised the clinically relevant possibility of residual or previously treated lymphomatous tissue serving as an ongoing source of antigenic stimulation. This provided a plausible mechanistic link between the remote NHL history and the delayed autoimmune presentation, although the absence of tissue biopsy precluded definitive histopathologic confirmation. The CT scan is shown in Figure 3.

Overall, the constellation of findings was consistent with warm AIHA (wAIHA), possibly complicated by iron overload due to chronic hemolysis.

## Discussion

This case highlights the potential for delayed-onset wAIHA in patients with a remote history of lymphoproliferative malignancy, a clinical scenario that remains rare but is increasingly recognized in the literature [1,2,7]. AIHA is most commonly associated with active or relapsed lymphoma, where malignant B-cell clones directly drive autoantibody production against host erythrocytes. However, late presentations occurring years after sustained remission likely reflect persistent immune dysregulation, treatment-induced perturbations of the immune microenvironment, or failure of long-term B-cell tolerance mechanisms. AIHA can also arise idiopathically or secondary to autoimmune diseases, viral infections, and certain medications, making a thorough diagnostic evaluation essential to exclude alternative etiologies in all presenting cases. 

The diagnosis in this case was supported by clinical symptoms (fatigue, pallor, and dark urine), laboratory evidence of hemolysis (elevated LDH, indirect hyperbilirubinemia, reticulocytosis), and a positive direct Coombs test [3,4]. The immunofixation profile suggested a skewed immunoglobulin response, possibly reflecting underlying immune activation. The presence of mesenteric calcifications further raised the concern for residual or treated lymphoma contributing to immune dysregulation.

Initial management of wAIHA typically involves high-dose corticosteroids, which the patient had already received [4,6]. To achieve a rapid response for symptomatic anemia due to active hemolysis, a single dose of intravenous immunoglobulin (IVIG) was administered. The patient also required transfusion of four units of packed RBCs (PRBCs), and initiation of rituximab was deemed necessary for additional disease control [5,8]. Progressive clinical improvement and stabilization of hemoglobin levels indicated a partial therapeutic response.

Clinical course and interpretation

Laboratory trends further supported the diagnosis of wAIHA secondary to immune dysregulation, potentially associated with residual lymphoma activity or ongoing immune activation. The rising reticulocyte count reflected an appropriate marrow response, whereas the decreasing LDH activity suggested a reduction in hemolytic activity with treatment. Fluctuations followed by gradual improvement in hemoglobin levels further corroborated the partial response to corticosteroids and IVIG, underscoring the importance of continued monitoring and individualized management.

This case highlights that residual structural and immunologic sequelae of lymphoproliferative malignancy may persist long after apparent clinical remission, with the potential to manifest as serious and life-threatening autoimmune complications requiring prompt recognition and aggressive multidisciplinary management.

Final recommendations

Patients with a history of NHL require structured long-term surveillance programs incorporating periodic cross-sectional imaging and comprehensive hematologic assessments, even after many years of sustained clinical remission, given the potential for delayed autoimmune complications to emerge decades after successful treatment. Clinicians should maintain a high index of suspicion for AHA in any patient presenting with unexplained anemia and a previous history of lymphoma, regardless of the duration of remission, as the temporal dissociation between lymphoma treatment and AIHA onset can be misleadingly reassuring. Serial laboratory monitoring - including hemoglobin trajectory, reticulocyte count, LDH, indirect bilirubin, and haptoglobin - remains an indispensable component of both diagnostic evaluation and ongoing disease management, enabling early detection of hemolytic activity and timely assessment of response to immunosuppressive therapy. Further dedicated research is warranted to elucidate the precise pathophysiological mechanisms linking residual or previously treated lymphomatous tissue to the development of late autoimmune phenomena, with the ultimate goal of identifying reliable biomarkers that could facilitate earlier detection of at-risk individuals before clinically significant hemolysis develops. Finally, a multidisciplinary approach integrating the expertise of hematology, oncology, and, where appropriate, immunology and rheumatology specialists is strongly recommended to ensure comprehensive evaluation, individualized treatment planning, and optimized long-term outcomes in this complex and underrecognized patient population.

## Conclusions

This case underscores the critical importance of sustained long-term vigilance in patients with a previous history of NHL, even those who have maintained complete clinical remission for a decade or more. AHA represents a potentially life-threatening delayed complication that may emerge years after successful lymphoma treatment, driven by persistent immune dysregulation, residual subclinical disease, or treatment-induced perturbations of the immune microenvironment, predisposing susceptible individuals to late autoantibody production. The severity of hemolysis in this patient, requiring multiple RBC transfusions and escalation to rituximab after an inadequate response to corticosteroids and IVIG, highlights that delayed-onset wAIHA can follow an aggressive clinical course, necessitating rapid diagnosis and timely immunosuppressive escalation. Cross-sectional imaging demonstrating mesenteric calcifications provided structural clues to possible residual or previously treated lymphomatous tissue that may have contributed to chronic immune activation. In contrast, immunologic markers - including immunoglobulin profiling, direct antiglobulin testing, and serial monitoring of LDH, haptoglobin, reticulocyte count, and indirect bilirubin - were essential for diagnosis, therapeutic guidance, and response assessment. Ultimately, this case supports structured long-term follow-up in NHL survivors incorporating periodic hematologic evaluation, immunologic profiling, and imaging to ensure early detection and management of delayed autoimmune complications before progression to hemodynamic instability or life-threatening anemia.
